# Mesenchymal Stem Cells Sense the Toughness of Nanomaterials and Interfaces

**DOI:** 10.1002/adhm.202203297

**Published:** 2023-02-21

**Authors:** Lihui Peng, Carlos Matellan, Minerva Bosch‐Fortea, Jordi Gonzalez‐Molina, Matteo Frigerio, Stefan Salentinig, Armando del Rio Hernandez, Julien E. Gautrot

**Affiliations:** ^1^ Institute of Bioengineering Queen Mary University of London Mile End Road E1 4NS London UK; ^2^ Cellular and Molecular Biomechanical Laboratory Department of Bioengineering Imperial College London London SW7 2AZ UK; ^3^ School of Engineering and Materials Science Queen Mary University of London Mile End Road London E1 4NS UK; ^4^ Department of Chemistry University of Fribourg Chemin du Musée 9 Fribourg 1700 Switzerland

**Keywords:** 2D nanomaterials, liquid–liquid interface, protein nanosheet, self‐assembly, stem cells, toughness

## Abstract

Stem cells are known to sense and respond to the mechanical properties of biomaterials. In turn, cells exert forces on their environment that can lead to striking changes in shape, size and contraction of associated tissues, and may result in mechanical disruption and functional failure. However, no study has so far correlated stem cell phenotype and biomaterials toughness. Indeed, disentangling toughness‐mediated cell response from other mechanosensing processes has remained elusive as it is particularly challenging to uncouple Youngs' or shear moduli from toughness, within a range relevant to cell‐generated forces. In this report, it is shown how the design of the macromolecular architecture of polymer nanosheets regulates interfacial toughness, independently of interfacial shear storage modulus, and how this controls the expansion of mesenchymal stem cells at liquid interfaces. The viscoelasticity and toughness of poly(l‐lysine) nanosheets assembled at liquid‐liquid interfaces is characterised via interfacial shear rheology. The local (microscale) mechanics of nanosheets are characterised via magnetic tweezer‐assisted interfacial microrheology and the thickness of these assemblies is determined from in situ ellipsometry. Finally, the response of mesenchymal stem cells to adhesion and culture at corresponding interfaces is investigated via immunostaining and confocal microscopy.

## Graphical abstract

Mesenchymal stem cells sense the toughness of polymer nanosheets assembled at liquid–liquid interfaces. The self‐assembly of poly(l‐lysine) and pentafluorobenzoyl chloride results in nanomaterials with interfacial toughness varying over orders of magnitudes. Stem cells spreading at these interfaces can fracture brittle interfaces but not those with the highest toughness, equivalent to that of steel.

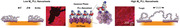

## 1 Introduction

The mechanical properties of biomaterials have a significant impact on a wide range of cell phenotypes, from the regulation of cell spreading and cell proliferation to the modulation of fate decision.^[^ ^1^, ^2^, ^3^ ^]^ In addition to cell response to the stiffness of their extra cellular environment, cells sense other mechanical features of their matrix, such as viscoelasticity^[^ ^4^, ^5^, ^6^ ^]^ and anisotropy.^[^ ^7^, ^8^ ^]^ These mechanical properties combine with ligand density,^[^ ^9^ ^]^ nanoscale deformation,^[^ ^10^ ^]^ matrix remodeling,^[^ ^11^ ^]^ and other biochemical cues to trigger and regulate mechanosensing pathways.^[^ ^2^, ^12^ ^]^ Such sensing involves molecular force sensors,^[^ ^13^, ^14^ ^]^ directly enabling the probing of nanoscale mechanical properties of the cell microenvironments.^[^ ^15^ ^]^ For example, cells have been found to respond directly to the local ligand density^[^ ^16^, ^17^ ^]^ and to rearrange their local microenvironment, resulting in the regulation of cell spreading and tissue or organoid development.^[^ ^11^, ^18^ ^]^ In turn, mechanosensing processes, combined to cell contractility, regulate tissue formation, remodeling and function.^[^ ^19^, ^20^ ^]^ Poor control of these parameters may result in the fracture and failure of biomaterials and interfaces, and the associated impact on tissue repair.^[^ ^21^, ^22^, ^23^ ^]^ However, little is known of the direct impact of materials toughness on cell phenotype, owing to the difficulty of uncoupling the toughness from other mechanical and physical parameters, at the cell scale and in a range relevant to cell‐mediated contractile forces.

The importance of local mechanical properties of materials is clearly illustrated by the ability of cells to adhere, spread and proliferate at the surface of low viscosity liquids.^[^ ^24^, ^25^, ^26^, ^27^, ^28^, ^29^ ^]^ Indeed, it was demonstrated that fibroblasts, epithelial cells such as HaCaTs and keratinocytes, and mesenchymal stromal cells can proliferate at the surface of fluorinated oils, providing a mechanically strong protein nanosheet formed at corresponding liquid‐liquid interfaces. This enabled the formation of cell colonies at the surface of low viscosity liquid substrates that were as spread and dense as those formed on rigid tissue culture plastic and sustained the preservation of stem cell phenotypes,^[^ ^30^, ^31^, ^32^ ^]^ despite the ultra‐weak bulk mechanical properties of underlying substrates.

The impact that polymer and protein self‐assembly has on liquid–liquid interfacial properties, including surface tension, interfacial pressure, and viscosity is well established.^[^ ^33^, ^34^ ^]^ However, the impact of chemical and structural parameters of corresponding macromolecules on interfacial mechanics, and in turn cell spreading and phenotype at liquid interfaces, remain poorly understood. In this respect, protein‐stabilized interfaces have been shown to display a broad range of interfacial mechanics and fluidity, resulting in the regulation of emulsion stability and associated formulations.^[^ ^35^, ^36^ ^]^ However, interfaces enabling the control of nanoscale mechanics and bioactivity, including cell adhesiveness, remain elusive.

In contrast to the toughness of a broad range of biomaterials, composites, ceramics and hydrogels, the interfacial toughness of structured liquid–liquid interfaces has not been investigated. Indeed, strategies enabling the toughening of materials have received significant attention, for example to design bioceramics and nacre‐like biomimetic materials, or for the design of tough elastomers and hydrogels.^[^ ^37^, ^38^, ^39^ ^]^ A number of key concepts have been proposed for such design, ranging from limiting defects in materials that may contribute to stress accumulation and fracture propagation, to energy dissipative mechanisms that can significantly limit fracture propagation.^[^ ^40^, ^41^, ^42^ ^]^ Hence hydrogels based on interpenetrated networks of soft and more rigid polymers, or the introduction of physical crosslinks that may contribute to energy dissipation, or the design of multiscale composites, have been proposed to create novel tough biomaterials.^[^ ^40^, ^43^, ^44^ ^]^ Similar concepts can be applied to 2D networks and the stabilization of liquid‐liquid interfaces, but such design remains to be established. Although interfacial dilatational rheology has been systematically applied to the study of interfacial mechanics, few works explore plasticity and fracture mechanisms in such context.^[^ ^45^, ^46^, ^47^ ^]^ Interfacial shear rheology is particularly suitable for quantifying fracture properties of liquid‐liquid interfaces, but this remains unexplored.

Poly(l‐lysine) (PLL) is a polycationic polymer that has been widely used for the functionalization of a broad range of interfaces, enabling the direct coupling of other macromolecules and bioactive moieties as well as the adsorption of extra‐cellular matrix proteins. It was found to result in the formation of particularly stiff nanosheets at liquid‐liquid interfaces, when assembling in the presence of reactive co‐surfactants such as pentafluorobenzoyl chloride (PFBC)^[^ ^31^ ^]^ and, in turn, maintain the preservation of stemness and long term expansion of mesenchymal stem cells (MSCs).^[^ ^30^ ^]^ However, little is known about the structural parameters governing the nanoscale mechanics of corresponding polymer/co‐surfactant assemblies. In this study, we examine the role of molecular weight on self‐assembly and interfacial mechanical properties of PLL nanosheets. We show that the molecular weight of PLL regulates interfacial toughness, resulting in interfaces displaying toughness comparable to that of steel, and enabling to resist cell‐mediated contractile forces, for the formation of large and dense colonies.

## 2 Results and Discussion

**Figure 1:**
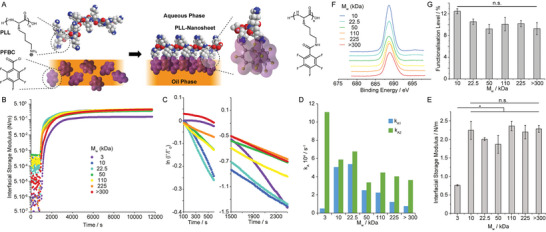
Impact of molecular weight on PLL nanosheet assembly at liquid–liquid interfaces. A) Molecular structure of PLL nanosheets and proposed resulting architecture. B) Evolution of the interfacial shear storage modulus of PLL nanosheets forming at Novec 7500‐water interfaces (Novec 7500 containing 10 µg mL^−1^ PFBC; aqueous solution is PBS with pH adjusted to 10.5; strain of 10^−3^ rad and 0.1 Hz). PLL with different M_w_ (3, 10, 22.5, 50, 110, 225, and >300 kDa) was introduced (after 900 s of equilibration) to make a final solution with a concentration of 100 µg mL^−1^. C) Corresponding ln(Γ(*t*)/Γ_0_) plots at two different time intervals following protein injection. D) Adsorption rate constants extracted from corresponding linear fits. E) Interfacial storage moduli as a function of *M*_w_ of PLL, measured from frequency sweeps at a strain of 10^−3^ rad and 0.1 Hz. Error bars are s.e.m.; *n* = 3. F) XPS spectra (F 1s) obtained for nanosheets generated with PLL with different *M*_w_. G) Functionalization levels quantified from corresponding XPS data (error bars are s.e.m.; *n* = 3). One‐way ANOVA; n.s., non‐significant; *p < 0.05.

PLL assembles into stiff nanosheets at liquid–liquid interfaces when combined with reactive co‐surfactants such as PFBC (**Figure** **1**A). To investigate the adsorption and interfacial mechanical properties of nanosheets assembled from PLL with different *M*_w_, we used interfacial rheology (a du Noüy ring positioned at the liquid–liquid interface, coupled to a DHR3 rheometer). We first examined the impact of *M*_w_ on the adsorption of PLL at a fluorinated oil (Novec 7500)‐aqueous (phosphate buffered saline, PBS) interface (Figure 1B). After equilibration of the system, PLL with different *M*_w_ was injected and the evolution of the interfacial shear moduli was monitored as a function of time. After a rapid initial increase, interfacial storage moduli gradually levelled within a range of 0.5–2.5 N m^−1^. The kinetics of adsorption was found to depend on the molecular weight of PLL (Figure 1C). To quantify associated kinetics, we applied a Langmuir first order model,^[^ ^48^ ^]^ assuming that interfacial shear moduli reflected the surface coverage at corresponding liquid–liquid interfaces. Adsorption traces were fit to the resulting equation:

(1) $$\ln\frac{\left(\Gamma_{f}-\Gamma \left(t\right)\right)}{\Gamma_{f}}=-k_{A}t$$

where Γ(*t*) and Γ_f_ are the surface coverage of PLL at time *t* and equilibrium and *k*_A_ is the adsorption rate constant (see Experimental Section). Our data was fitted over two separate early stages of the adsorption profiles, 100–600 s and 1500–2500 s (affording two rate constants, *k*_A1_ and *k*_A2_, respectively; Figure 1C,D). Although most traces fit a linear relationship, some deviation was clearly observed for the lowest molecular weight PLL tested (3 kDa). A gradual decrease in both rate constants was observed as a function of PLL *M*_w_, consistent with the expected impact of steric and coulombic hindrance associated with polyelectrolyte adsorption. However, *k*_A1_ measured for 3 kDa PLL was significantly lower, presumably due to the difficulty of achieving a percolated network at the liquid–liquid interface with low molecular weight molecules. In contrast, PLL chains with higher *M*_w_ can bridge across isolated adsorption islands more readily. They may be expected to form a percolated network at early time points, following which stage the interfacial storage modulus may better reflect changes in polymer surface densities.

Despite differences in adsorption kinetics, the ultimate (equilibrium) interfacial storage modulus of PLL interfaces was strikingly similar at different *M*_w_ (Figure 1E). The only interfaces displaying slightly lower interfacial shear storage moduli were those formed with 3 kDa PLL (0.76 N m^−1^, compared to 2.0–2.3 N m^−1^ for higher *M*_w_). To examine whether assembled nanosheets were associated with changes in PLL adsorption densities, we characterized the abundance of PLL at corresponding interfaces, using tagged polymers and fluorescence microscopy (Figure S1, Supporting Information). This indicated comparable levels of polymer adsorption at liquid‐liquid interfaces, independent of *M*_w_. Similarly, we characterized variations in the degree of functionalization level of PFBC achieved on nanosheets from PLL with varying *M*_w_. To test this hypothesis, we characterized the atomic composition of PLL nanosheets by XPS (Figure 1F,G). Fluorination levels and associated PFBC functionalization levels were comparable for all nanosheets, independent of the molecular weight of the PLL used. The thickness of PLL nanosheets, characterized by neutron reflectometry in situ, was found to be in the range of 6–10 nm.^[^ ^49^ ^]^ Therefore, we estimate the equivalent bulk shear modulus of materials that would be formed of PLL nanosheets to be in the range of 200–300 MPa. Such high stiffness implies the formation of a continuous rigid phase, which we propose is rich in rigid aromatic moieties able to aggregate via the formation of J‐stacks (Figure 1A), owing to the strong quadripolar nature of fluorinated aromatics.^[^ ^50^ ^]^ PLL nanosheets displaying comparable functionalization with pentafluorobenzoate moieties would therefore be expected to display comparable interfacial storage moduli.

**Figure 2:**
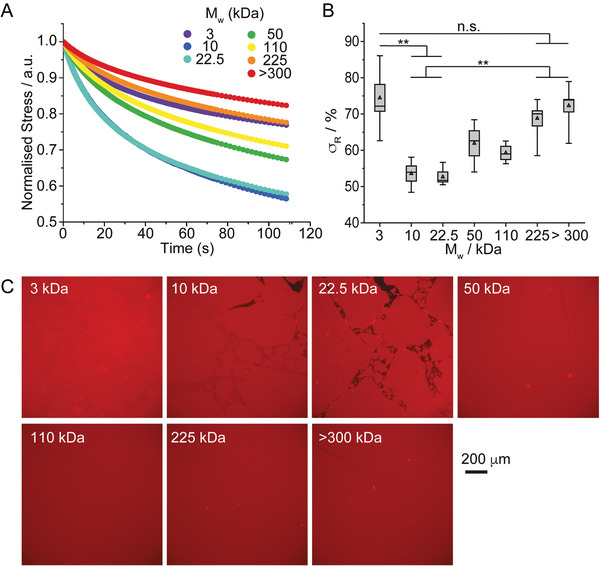
Interfacial viscoelasticity is controlled by the molecular weight of PLL. A) Representative stress relaxation profiles of nanosheets assembled from PLL with different molecular weights (strain: 10^−3^ rad). B) Corresponding stress retentions *σ*_R_ extracted from the corresponding fits. Error bars are s.e.m.; *n* ≥ 3. C) Epifluorescence microscopy images of PLL nanosheets assembled with PLL with a range of *M*_w_ (all tagged with Alexa Fluor 488). Detail of interfaces: Novec 7500 containing 10 µg mL^−1^ PFBC; aqueous solution is PBS with pH adjusted to 10.5; PLL with different *M*_w_ (3, 10, 22.5, 50, 110, 225, and >300 kDa) at a final concentration of 100 µg mL^−1^. One‐way ANOVA; n.s., non‐significant; **p < 0.01.

Considering the important role of viscoelasticity in the regulation of cell adhesion, migration, and fate decision,^[^ ^6^ ^]^ we next examined how the molecular weight of PLL impacted on the viscoelastic profile of nanosheets. Indeed, the interfacial storage modulus of PLL nanosheets displayed some frequency dependency associated with a clear viscoelastic response (Figure S2, Supporting Information). To characterize further viscoelasticity at PLL interfaces, we carried out interfacial stress relaxation experiments, using a double exponential decay model. Upon application of a defined strain (typically 0.1–1%), PLL nanosheets displayed apparent stress relaxation, with ultimate stress retention *σ*_R_ in the range of 55–75% (**Figure** **2**A,B). Apart from nanosheets formed from 3 kDa PLL, our data indicated a gradual increase in the *σ*_R_, and therefore elasticity, as a function of increasing molecular weight.

This trend was surprising, considering the absence of change in interfacial storage modulus observed (Figure 1E) and, to gain further insight into this behavior, we imaged corresponding liquid‐liquid interfaces after formation of nanosheets assembled from tagged PLL with different *M*_w_ (Figure 2C). Although surface densities of PLL were found to be comparable (Figure S1, Supporting Information), the morphology of interfaces differed widely, dependent on PLL *M*_w_. Whereas at low molecular weight interfaces were apparently formed of fragmented nanosheets, they appeared homogenous and continuous over very large distances at higher molecular weights, with domains exceeding several millimeters. The transition to such behavior was in the range of 50 kDa. Interestingly, although domains were clearly visible for the lowest molecular weight PLL tested (3 kDa), they remained tightly packed and no gap between such domains could be observed by microscopy. This may explain the high *σ*_R_ measured for nanosheets formed with 3 kDa PLL and suggests that the viscous behavior observed in PLL nanosheets results from inter‐domain relaxation. Such behavior would indeed result in better retention of elastic properties in stress‐relaxation experiments (constant deformations), whereas creep resistance would be less sensitive to such small domain relaxation as deformations would not be prevented.

We next carried out interfacial creep experiments, again using the 6‐element Burger's model to quantify associated data (Figure S3A,B, Supporting Information). At low interfacial stress (1 mN m^−1^), our data indicated a classic viscoelastic response (Figure S3C, Supporting Information), with no significant change in the main shear modulus *G*_0_ as a function of PLL *M*_w_, although *G*_1_ and *G*_2_ did increase slightly for PLL with *M*_w_ > 300 kDa (Figure S3D, Supporting Information). As in the case of stress relaxation experiments, the viscous component was found to increase significantly at intermediate *M*_w_ (Figure S3E, Supporting Information). However, at higher applied stress (5 and 10 mN m^−1^), failure was clearly observed, depending on *M*_w_: interfaces formed with 50 kDa PLL failed at 10 mN m^−1^, whereas those formed with 3 kDa PLL failed already at 5 mN m^−1^ (Figure S3C, Supporting Information). Hence nanosheet fracture and relaxation seemed to be strikingly impacted by the molecular weight of PLL.

**Figure 3:**
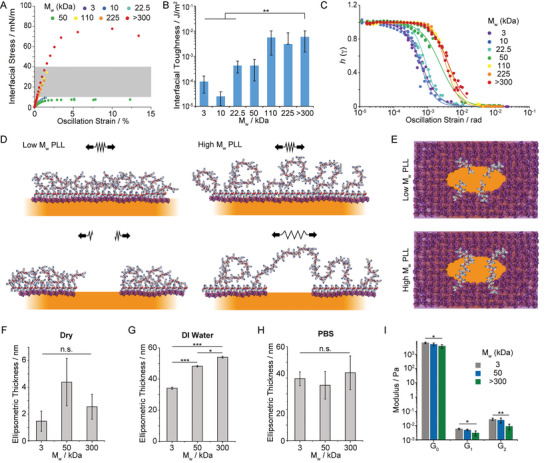
The nanoscale architecture of PLL nanosheets controls interfacial toughness. A) Representative shear stress‐strain curves extracted from amplitude sweep experiments (frequency of 0.1 Hz). The grey area shaded correspond to the range of interfacial stresses expected to be exerted by mature focal adhesions. B) Summary of interfacial toughness calculated from the corresponding shear stress–strain profiles. (error bars are s.e.m.; *n* ≥ 3). C) Damping functions calculated from strain sweeps. The trend lines correspond to fits with the Soskey–Winter model. D,E) Proposed model of nanosheet fracture, depending on the molecular weight of PLL chains (D, side view; E, top view; only some chains localized at the fracture line are represented for improved visualization). Ellipsometric thickness of selected nanosheets determined F) dry, G) in deionized (DI) water, and H) PBS. Nanosheets were transferred to silicon substrates using a Langmuir–Blodgett liquid–liquid trough, prior to characterization. Error bars are s.e.m.; *n* = 3. I) Summary of magnetic‐tweezer assisted interfacial microrheology data (shear moduli *G*_0_, *G*_1_, and *G*_2_ extracted using the six elements Burger's model). Detail of interfaces: Novec 7500 containing 10 µg mL^−1^ PFBC; aqueous solution is PBS with pH adjusted to 10.5; PLL with different *M*_w_ (3, 10, 22.5, 50, 110, 225, and >300 kDa) at a final concentration of 100 µg mL^−1^. Error bars are s.e.m.; *n* ≥ 7. One‐way ANOVA; n.s., non‐significant; *p < 0.05; **p < 0.01; ***p < 0.001.

Interfacial oscillatory rheology in amplitude sweeps was next carried out. PLL nanosheets displayed broad linear regions at low oscillation amplitudes, whereas significant thinning and non‐linearity were observed at higher amplitudes. Such phenomenon is typical of the viscoelastic profile of concentrated polymer solutions and soft physically crosslinked polymer networks^[^ ^51^, ^52^ ^]^ and was previously reported for other liquid‐liquid interfaces stabilized by protein surfactants.^[^ ^36^ ^]^ The toughness apparent from these amplitude sweeps, characterized from corresponding strain–stress traces, varied markedly depending on the molecular weight of the PLL forming the nanosheet (**Figure** **3**A). We extracted interfacial toughness from these measurements, confirming a threshold of 50 kDa above which the nanosheets were significantly reinforced (Figure 3B), in agreement with the nanosheet morphologies observed by fluorescence microscopy. Analysis of the damping function *h*(*γ*) associated with such non‐linear viscoelastic profiles confirmed this threshold, with a significant shift in the position of the amplitude at which decay of the function and damping are observed (Figure 3C). In concentrated polymer solutions and at liquid–liquid interfaces, the damping functions are typically observed to collapse on the same profile, overlapping with the Soskey–Winter model.^[^ ^53^ ^]^ In contrast, the striking shift in the damping function observed for *M*_w_ above 50 kDa, with overlapping functions for *M*_w_ > 50 kDa, implies a different mechanism for strain‐induced softening. Indeed, although parameters associated with the Soskey–Winter damping model are not formally linked to molecular architectures (e.g., molecular weight, degree of crosslinking), the damping function is typically considered to reflect the mechanism of disruption of entanglements and physical bonds forming a polymer solution or network.^[^ ^36^, ^52^ ^]^ Therefore, the shift in damping function observed, by one order of magnitude in oscillation strain, is proposed to reflect a switch in domain relaxation and remodeling, from inter‐domain to intra‐domain rearrangement.

To further explore the mechanism of fracture mechanics and relaxation of PLL nanosheets with different *M*_w_, we characterized the thickness of nanosheets and their swelling. Nanosheets were transferred from corresponding liquid‐liquid interfaces to mica and silicon substrates via Langmuir‐Blodgett transfer. Tagged nanosheets were transferred to mica substrates to confirm via fluorescence imaging that large macroscopic nanosheets covering the surface of the target substrates could be transferred (Figure S4, Supporting Information). Nanosheets transferred to silicon substrates were then characterized by ellipsometry (Figure 3F–H). Dry nanosheets displayed thicknesses in the range of 1–4 nm. In deionized water, the hydrophilic phase of nanosheets increasingly swells, to 30–50 nm, depending on PLL *M*_w_. In contrast, in PBS the swelling of all nanosheets was comparable and reduced compared to deionized water. Such ionic strength‐dependent behavior is expected from polyelectrolytes tethered to interfaces^[^ ^54^, ^55^, ^56^ ^]^ and increased hydrodynamic diameter associated with PLL chains of increasing *M*_w_.^[^ ^57^, ^58^ ^]^ To further confirm these results, in situ ellipsometry was carried out directly at liquid‐liquid interfaces (Figure S5, Supporting Information). Swollen ellipsometric thicknesses of 14.7, 15.8, and 21.9 nm were extracted from those data, confirming the increase in thickness measured for nanosheets transferred to solid substrates. Differences with the swollen thicknesses reported in Figure 3G/H likely reflect the formation of folds in the nanosheets when transferred to solid substrates.

In addition, we characterized the nanoscale mechanical properties of the soft hydrophilic phase of PLL nanosheets via magnetic‐tweezer assisted interfacial micro‐rheology. Epoxylated magnetic particles (4.5 µm) were allowed to adhere to PLL nanosheets, prior to applying a 30 s force pulse via a magnetic tweezers device (Figure S6, Supporting Information). Bead trajectories were monitored (50 frames per s), the creep profile associated with such stimulation was then modelled using a 6‐element Burger's model and the associated shear moduli were quantified (Figure 3I). In contrast to the macroscopic interfacial rheology data obtained (Figure 1E; Figure S3, Supporting Information), the moduli of PLL interfaces were found to decrease as a function of molecular weight. Together with our ellipsometry data, this suggests that the hydrated, swollen soft phase of PLL nanosheets is increasingly soft and stretchable at high PLL *M*_w_. In turn, this soft phase is able to bridge across fracture cracks, dissipate local energy and reinforce the brittle PFBC‐rich hard phase of PLL nanosheets (Figure 3D,E), in an analogous manner to polymer‐reinforced composites^[^ ^42^, ^59^, ^60^ ^]^ and engineered tough hydrogels.^[^ ^40^, ^41^ ^]^

**Figure 4:**
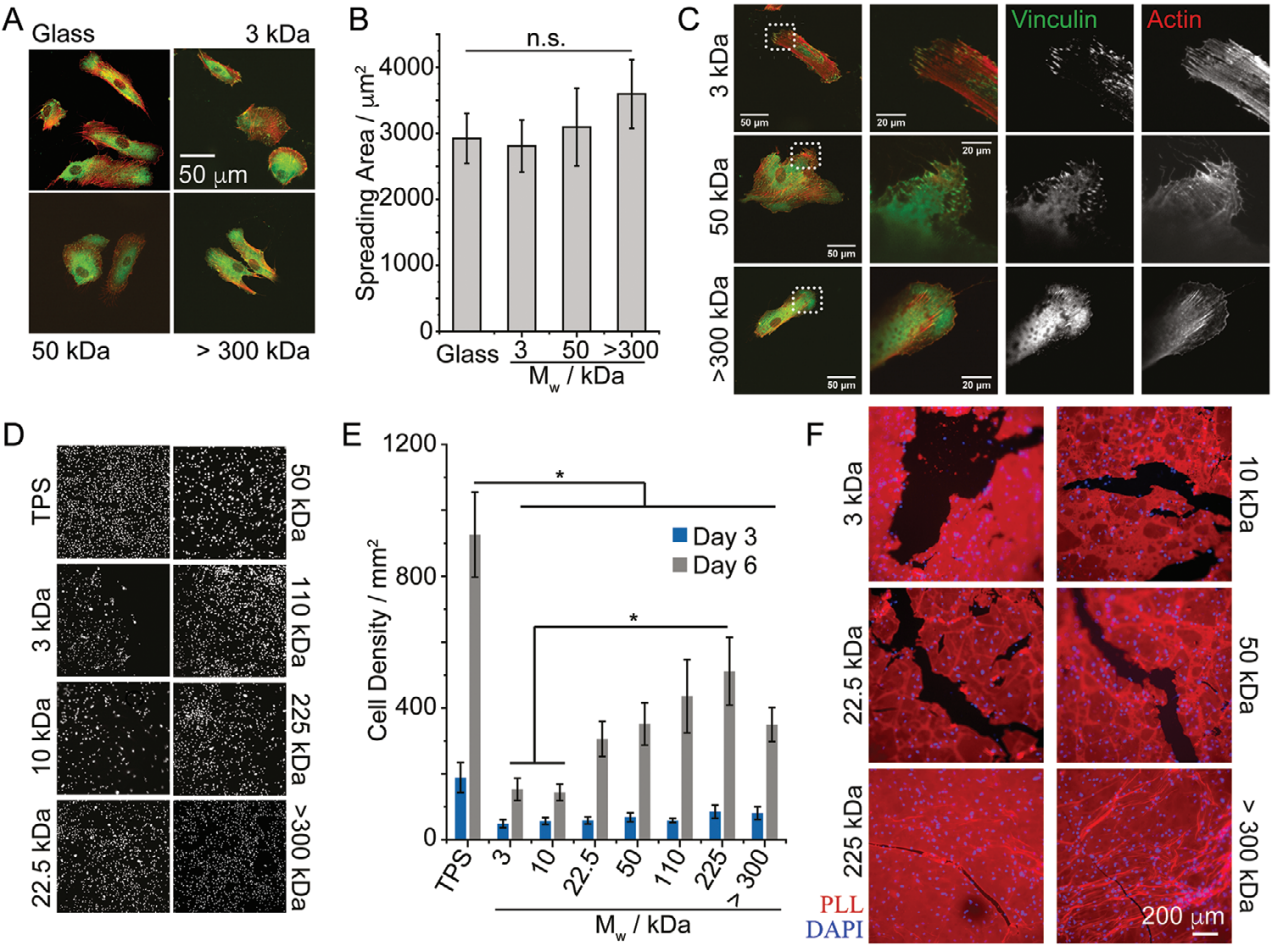
Stem cell expansion at liquid interfaces correlates with interfacial toughness. A,B) Impact of PLL molecular weight on cell spreading at Novec 7500 interfaces stabilized by corresponding nanosheets. C) Confocal microscopy images of MSCs spreading (after 24 h) on PLL/FN functionalized Novec 7500 interfaces. Zoom‐in correspond to the dotted boxes. D,E) MSC expansion at PLL‐stabilized Novec 7500 interfaces (D, representative nuclear stainings). F) Highly confluent MSCs remodel and fracture PLL/FN nanosheets assembled at the surface of Novec 7500. Epifluorescence microscopy images of PLL nanosheets 24 h after seeding MSCs at 200 000 cell/well (left). Red, PLL; blue, nuclei. Detail of interfaces: Novec 7500 containing 10 µg mL^−1^ PFBC; aqueous solution is PBS with pH adjusted to 10.5; PLL with different *M*_w_ (3, 10, 22.5, 50, 110, 225, and >300 kDa) at a final concentration of 100 µg mL^−1^. Error bars are s.e.m.; *n* ≥ 4. One‐way ANOVA; n.s., non‐significant; *p < 0.05.

We next examined how the toughness of PLL nanosheets may impact on stem cell adhesion and proliferation at liquid interfaces. Mesenchymal stem cells (MSCs) were allowed to adhere to fibronectin ‐coated PLL nanosheet‐stabilized liquid–liquid interfaces and their spreading was characterized by immunostaining and confocal microscopy (**Figure** **4**A,B). Cells assembled a structured actin cytoskeleton on liquid substrates, despite the fluidity and low viscosity of the underlying substrate (Novec 7500). Morphological analysis revealed no significant difference between cells adhering to PLL nanosheets with different *M*_w_ and cells adhering to rigid glass coverslips coated with PLL and fibronectin. Cell circularity and aspect ratio were also found to be comparable (Figure S7A,B, Supporting Information). Similarly, MSCs formed focal adhesions located at the end of stress fibers on all PLL nanosheets, independent of the PLL *M*_w_ (Figure 4C). No differences in the number of focal adhesions formed could be observed, but a slight increase in the size of focal adhesions formed was observed between cells spreading on nanosheets based on >300 kDa PLL, compared to lower molecular weight PLL (3 and 50 kDa; see Figure S7C,D, Supporting Information).

Therefore, consistent with the impact of hydrogels and biomaterials mechanics,^[^ ^61^, ^62^ ^]^ MSC adhesion to PLL nanosheet‐stabilized interfaces with comparable interfacial storage moduli had no significant impact on cell adhesion and spreading. However, the proliferation of MSCs was significantly impacted by the molecular weight of PLL assembling nanosheets and resulting interfacial toughness (Figure 4D,E). This was not associated with any change in cell viability, which remained high and comparable on all substrates, although we noted a slight increase in cytotoxicity for the lowest *M*_w_ PLL at early time points (Figures S8,S9, Supporting Information). The abundance of fibronectin deposited at PLL nanosheet interfaces was also comparable (Figures S10,S11, Supporting Information), in agreement with the similar spreading observed and the observation of focal adhesions and structured actin cytoskeleton on the different PLL interfaces tested. Instead, we propose that cells spreading on nanosheets assembled from lower *M*_w_ PLL sense the toughness of corresponding interfaces. Indeed, gaps in cell coverage were observed in cultures at nanosheets formed from low and intermediate *M*_w_ PLL. Hence, cell‐mediated forces are proposed to locally fracture PLL nanosheets, or to extend shear induced fractures occurring during substrate preparation, leading to local relaxation of the 2D network and gradual reduction of the elasticity of the network. In turn, fracture and the associated occurrence of gaps within these networks change the adhesive landscape: through the limitation of the cell adhesive area available and by generating softer areas that are not tethered and therefore may not provide as stiff and robust environment to sustain spreading.

The impact of such a process on cell proliferation was examined via Ki67 immunostaining and fluorescence microscopy. After 48 h of culture, MSCs displayed levels of Ki67 expression comparable to high molecular weight PLL nanosheets to control glass substrates (Figure S12, Supporting Information). This is in good agreement with cell densities measured and with previous observations that MSCs proliferated at comparable rates at PLL nanosheet interfaces compared to tissue culture plastic.^[^ ^30^ ^]^ In contrast, on low molecular weight PLL nanosheets (3 kDa), the density of Ki67 positive cells was reduced, compared to that observed on high molecular weight PLL (>300 kDa). Therefore, these data suggest that the impact of PLL molecular weight and associated interfacial toughness on MSC proliferation results from a combination of limitation of cell adhesive surface area and regulation of cell cycling.

To investigate the ability of cells to fracture nanosheets, depending on their molecular weight, MSCs were cultured on tagged PLL nanosheets, prior to imaging after 24 h of culture (Figure S13A, Supporting Information). Cells on low molecular weight PLL nanosheets (3 and 50 kDa) can be seen to fold and fracture nanosheets in multiple areas, resulting in large aggregates of PLL material accumulating. In contrast, on high molecular weight PLL nanosheets (>300 kDa), few areas displayed folded nanosheet morphologies and aggregates, with cells adhering to nanosheets without inducing significant defects. In addition, when cultured in the presence of blebbistatin (20 µm), nanosheet disruption was prevented. Cells could be seen to spread on all interfaces (Figure S13B, Supporting Information), as was previously observed in the case of keratinocytes spreading on intermediate molecular weight PLL nanosheets (corresponding to 50 Da PLL).^[^ ^31^ ^]^ Finally, to better evidence cell‐mediated fracture of PLL nanosheets, we seeded MSCs at high densities at nanosheet‐stabilized interfaces and characterized the morphology of resulting cultures 24 h after seeding. Clearer gaps could be seen in high density cultures seeded on low *M*_w_ PLL nanosheets (Figure 4F) and these gaps were clearly associated with fractures in PLL nanosheets. This suggests that local fracture in nanosheets, together with concerted contractile forces, further extending such defects to dimensions spanning several hundreds of microns, results in these gaps in dense cell cultures.

## 3 Discussion and Perspective

Overall, our data demonstrate that cells can directly sense the nanoscale toughness of interfaces to which they adhere and, despite developing mature adhesions at early time points, can mechanically disrupt their adhesive landscape, leading to retraction of adhesive areas and reduction in cell expansion. With forces exerted by cells in the range of 1–50 nN per adhesion, and focal adhesions displaying cross‐sections of 500 nm to 2 µm, the equivalent interfacial stress that can be expected to be transferred to nanosheets lies within the range of 5.10^−4^–0.1 N m^−1.^^[^ ^14^, ^63^, ^64^, ^65^, ^66^ ^]^ More specifically, in the case of MSCs, fully mature adhesions were found to generate stresses near 10–40 mN m^−1^ (maximum forces in the range of 20–40 nN, with adhesions 1–2 µm in cross‐section^[^ ^63^, ^64^ ^]^). This is a range between the ultimate interfacial stress that we measured for low and high *M*_w_ PLL nanosheets (Figure 3A). Therefore, the transition observed in interfacial toughness is proposed to overcome the maximum stress exerted by contractile cell adhesions, enabling to sustain nanosheet integrity over prolonged culture times.

Although the size of the nanosheet domains that are formed is in the micron‐range, it is worth pointing out that initial deformation and fracture must originate at the nanoscale, before crack propagation and ultimately micro‐ to macro‐scale fracture and domain formation. Such processes are typical of the fracture mechanics of other materials, including composites and hydrogels.^[^ ^37^, ^38^, ^41^ ^]^ In this respect, interfacial stress–strain data do indicate strains at break that are associated with deformations in the range of 10–150 µm, depending on the molecular weight of the PLL used. This also agrees with the micron‐scale deformations likely exerted by cells as they spread on corresponding interfaces, as evidenced by the folding of nanosheets in response to cell mediated contraction (Figure S13, Supporting Information). In addition, in agreement with fracture behavior typically accepted in other materials, the origin of failure and the scale at which initial network disruption is observed has to be at the nanoscale. Therefore, the schematics presented in Figure 3D is only representing the likely scenario we propose takes place at the molecular scale early in the failure process, leading to crack propagation as deformations over µm‐scales are sustained and large (tens to hundreds of µm) domains form.

The ability of cells to sense the mechanical properties of their environment is enabled by the reciprocal responses of the adhesion machinery (underpinned by integrin binding, actin assembly and contractility and mediated by adapter proteins such as talin and vinculin^[^ ^13^, ^67^ ^]^) and the nanoscale mechanics of corresponding interfaces.^[^ ^9^ ^]^ Deformation, strain stiffening and clustering, associated with the viscoelastic profiles of corresponding materials are integral elements sensed by cell adhesions and triggering downstream signaling. Our work proposes that the toughness of interfaces can further modulate such processes, not through the direct regulation of cell adhesion, but by defining a threshold above which interface and matrix remodeling lead to failure of the adhesive landscape, and as a result the retraction of cell adhered from associated areas. This concept is important to the engineering of biomaterials displaying significant mismatch in mechanical properties in bulk and at interfaces, as in the case of cell culture on liquid substrates and bioemulsions. It also underpins some of the processes occurring during matrix remodeling. For example, during the deposition of extra‐cellular matrix and its mechanical integration to pre‐existing biomaterials or tissues, or during tissue contraction in wound healing or tissue regeneration contexts. Further experiments may indicate how interfacial toughness, beyond the regulation of MSC proliferation, may also impact a broader range of phenotypes.

## 4 Experimental Section

### Interfacial Rheology

Interfacial rheology was carried out on a hybrid rheometer (DHR‐3) from TA Instruments fitted with a double wall ring (DWR) geometry and a Delrin trough with a circular channel. The double wall ring used for this geometry had a radius of 34.5 mm and the thickness of the Platinum–Iridium wire was 1 mm. The diamond‐shaped cross‐section of the geometry's ring provided the capability to pin directly onto the interface between two liquids and measure the interface properties without sub‐phase correction. 19 mL of the fluorinated oil (Novec 7500, ACOTA) pre‐mixed with pentafluorobenzoyl chloride (PFBCl, Sigma‐Aldrich) at desired concentrations was placed in the Delrin trough and the ring was lowered, ensuring contact with the surface, via an axial force procedure. The measuring position was set 500 µm lower than the contact point of the ring with the oil‐phase surface. Thereafter, 15 mL of the pH 10.5 PBS buffer was carefully syringed on top of the oil phase. Time sweeps were performed at a constant frequency of 0.1 Hz and a temperature of 25 °C, with a displacement of 1.0 × 10^−3^ rad to follow the formation of the protein layers at the interface. The concentration of poly(l‐lysine) (PLL) used for all rheology experiments were 100 µg mL^−1^ (with respect to aqueous phase volume), respectively. Before and after each time sweep, frequency sweeps (with a constant displacement of 10^−3^ rad) were conducted to examine the frequency‐dependent characteristics of the interface.

Before amplitude sweeps (with constant frequencies of 0.1 Hz) were carried out to ensure that the chosen displacement was within the linear viscoelastic region, stress relaxation was performed at 1% strain for 120 s. Considering the low moduli initially measured for pristine liquid–liquid interfaces (in the absence of protein and/or surfactant), viscous drag from both phases were not corrected. It was noted that although the interfacial shear moduli observed at liquid–liquid interfaces in the absence of protein or surfactant were expected to be considerably lower than those measured in our assay,^[^ ^68^, ^69^ ^]^ due to lack of viscous drag correction, they should not completely vanish and interfacial shear viscosity should be expected to persist, even with liquid–liquid interfaces that display very limited roughness (at the molecular scale). Damping functions were generated by normalizing strain sweep data to moduli measured at the lowest strain. To quantify interfacial toughness, corresponding stress‐strain plots were integrated. It was noted that these interfacial toughness data, extracted from such shear experiments, could not be quantitatively compared to toughness data extracted from tensile testing, even after integration of interfacial toughness to the thickness of nanosheets. Extrapolation of interfacial moduli and toughness to bulk shear moduli and toughness were carried out by integration of the corresponding interfacial modulus or toughness (assumed to be infinitely small in interfacial rheology) over the thickness of corresponding nanosheets (based on neutron reflectometry measurements,^[^ ^49^ ^]^ close to 10 nm).

### Data Analysis from Interfacial Stress Relaxation Experiments

Stress relaxation data from the 10th second onward when the stress relaxation started was plotted as stress against time in scatter in OriginPro, and fitted with double exponential decay fit, according to the following equation:

(2) $$\sigma =\sigma_{e}+\sigma_{1}\left(1-e^{-t/\tau_{1}}\right)+\sigma_{2}\left(1-e^{-t/\tau_{2}}\right)$$

In this equation, *σ* is the measured residual stress, *σ*_e_ is the elastic stress, and *σ*_1_ and *σ*_2_ are viscous relaxation components. The degree of stress retention (*σ*_r_) was calculated as:

(3) $$\sigma_{r}=\frac{\sigma_{e}}{\sigma_{e}+\sigma_{1}+\sigma_{2}}\times 100$$

### Interfacial Creep Experiments

For creep recovery experiments, a double wall Du Noüy Ring geometry (20 mm in diameter and 400 µm in thickness) and trough of corresponding dimensions were used. 3 mL of the fluorinated oil premixed with 10 µg mL^−1^ PFBC were placed in the Delrin trough and the ring was lowered, ensuring contact with the surface, via an axial force procedure. The measuring position was set 200 µm lower than the contact point of the ring with the oil phase surface. Thereafter, 4 mL of the pH 10.5 PBS buffer was carefully syringed on top of the oil phase. Time sweep was performed at a constant frequency of 0.1 Hz and a temperature of 25 °C, with a displacement of 10^−3^ rad to follow the formation of the protein layers at the interface. A 40 µL amount of 10 mg mL^−1^ (in DI water) PLL with molecular weight of interest was pipetted to the aqueous phase, making a final concentration at 100 µg mL^−1^. When the time sweep measurement was completed, the excess polymer solution was washed by diluting with PBS (pH 7.4) six times. The creep recovery experiment was then carried out by applying a 10^−3^, 5 × 10^−3^ or 10^−4^ Pa stress for a duration of 30 s, followed by a 30 s recovery at 0 stress. Due to creep ringing artefacts resulting from the coupling of the instrument inertia and sample elasticity in a stress controlled rheometer,^[^ ^46^, ^70^, ^71^ ^]^ smoothing of traces was applied in Origin and the creep results were fitted with a 6‐element modified Burger's model in MATLAB, according to the following equation:

(4) $$J=\frac{1}{G_{0}}+\frac{1}{G_{1}}\left(1-e^{-t/\tau_{1}}\right)+\frac{1}{G_{2}}\left(1-e^{-t/\tau_{2}}\right)+\frac{t}{\eta }$$

where *J* is the creep compliance, whereas *G*_0_, *G*_1_, *G*_2_, are the elastic and viscous shear moduli (two relaxation time components), and *τ*_1_, *τ*_2_, and *η* are the relaxation times and viscosity of the corresponding interfaces.

### Generation of Pinned Droplets

Glass samples were placed into a desiccator together with an open vial containing toluene (1 mL) and 30 µL trichloro (1H, 1H, 2H, 2H‐perfluorooctyl) silane (Sigma). The desiccator was placed under vacuum for 5 min and then left under reduced atmosphere but sealed overnight. After 24 h incubation, the glass slides were washed with ethanol and dried in air. The resulting hydrophobic glass slides were cut into 1×1 cm samples and placed into a 24‐well plate. After sterilization with 70% ethanol, samples were washed with PBS and filled with 1 mL PBS (with pH adjusted as indicated). 100 µL pinned droplets of fluorinated oil (Novec 7500) with the fluorinated co‐surfactant at desired concentrations were deposited on top of the submerged coated glass slide. 1 mL of 200 µg mL^−1^ PLL solution was pipetted into each well (final concentration of 100 µg mL^−1^) and left to incubate for 1 h. Samples were washed by successive dilution/aspiration with PBS (pH 7.4, 6 times). Fibronectin adsorption was carried out by adding 20 µL of a fibronectin solution (1 mg mL^−1^) into each well (final concentration: 10 µg mL^−1^), followed by incubation at room temperature for 1 h. Finally, samples were washed by dilution/aspiration with PBS (pH 7.4) four times and then with growth medium twice.

### Interfacial Creep Microrheology: Preparation of Samples

Fluorinated glass slides (prepared as above) were placed into a 3‐cm petri‐dish and covered with 5 mL of PBS (normally at pH 10.5 unless otherwise specified). Pinned droplets of 100 µL fluorinated oil with PFBCl at desired concentrations were deposited at the fluorinated surface, covering the entire substrate. 50 µL PLL solution (10 mg mL^−1^) was pipetted into the dish, making a final concentration of 100 µg mL^−1^. After 1 h incubation, the polymer solution was washed by sequential dilution/aspiration with PBS (pH 7.4) six times. Meanwhile, epoxylated dynabeads (4.5 µm, Dynabeads M‐450 Epoxy, Thermo Fisher) were diluted in PBS (100 times from stock), and 50 µL of the resulting suspension was homogeneously pipetted onto the dish and left to incubate for 15 min. The excess beads in the dish were removed by sequential dilution/aspiration with PBS (pH 7.4) six times, prior to the start of measurements.

### Magnetic Tweezer‐Operated Interfacial Creep Microrheology

Interfacial creep‐recovery experiments were performed using magnetic tweezers. Magnetic beads bound to the nanosheets were subjected to a force pulse with a magnitude of 6 nN (40 µm distance between the bead and the tip) and a duration of 30 s. Their trajectories were recorded for a total of 1 min per bead with a frame every 20 ms. Around 30 traces were obtained per dish, per experiment. Bead tracking was achieved by MATLAB and fitted with a 6‐element modified Burger's model (two Kelvin–Voigt elements in series with a Maxwell element, extended from a 4‐element model^[^ ^4^, ^72^ ^]^), according Equation (4) and defining parameters as previously described.^[^ ^49^ ^]^ For *G*_0_, *n* = 11, 8, 7 for low, medium, and high *M*_w_, respectively; for *G*_1_, *n* = 10, 8, 7 for low, medium, and high *M*_w_, respectively; for *G*_2_, *n* = 9, 8, 8 for low, medium, and high *M*_w_, respectively.

### Ellipsometry

The thickness of PLL nanosheets was determined using a J.A. Woollam *α*‐SE spectroscopic ellipsometer, after transfer from the corresponding liquid–liquid interfaces onto silicon substrates. Each sample was measured in three different positions, and experiments were carried out in triplicates. The data was fitted using a Cauchy model, with a single layer of polymer adsorbed to a silicon/silicon oxide interface. For in situ ellipsometry measurements at the liquid‐liquid interface, adsorption kinetics were carried out at the Brewster angle, as multi‐angle ellipsometry in rotating compensator mode between 40.0 and 50.0°, using an Accurion EP4 imaging ellipsometer (Accurion, Goettingen, Germany). The ellipsometer has a software‐controlled high‐precision goniometer with an angle of incidence range between 38° and 90° at an angle resolution of 0.001°. It is equipped with a 658 nm diode laser with 50 mW maximum power, a CCD camera as the detector (monochrome Gigabit Ethernet (GigE ) CCD‐Kamera, 1392×1040 pixels, 12 bits) as well as light‐guides for the assessment of liquid‐liquid interfaces. A 5× objective was used in this study, with a field‐of‐view of 0.8 mm and a lateral ellipsometric resolution of 4 µm). The temperature for all measurements was 20 °C. 10 µg mL^−1^ of PFBC solution in Novec 7500 was placed in the bottom compartment of a liquid/liquid trough (inner diameter 8 cm, outer diameter 10 cm). Optical guides were integrated to guide the light through the air‐liquid interface for reflection to only arise from the liquid/liquid interface. Subphase normal and surface were aligned with the virtual 0° angle of incidence and the intersection of the probing beam and the optical axis of the imaging arm, respectively. A pH 10.5 PBS solution was carefully deposited onto the subphase. The angular range was scanned for a minimum in‐camera signal, corresponding to the Brewster angle at which reflectivity of a 1 mm^2^ region of interest was monitored over 30 min, to ensure a stable measurement. 0.45 mL of PLL solution (three different molecular weights, 3, 50, and >300 kDa), corresponding to a 100 µg mL^−1^ solution in PBS, were injected in multiple locations to improve homogeneity. The change in reflectivity was monitored over time until signal saturation. The model fitting was performed with the Accurion EP4 model software package. The adsorption was modelled as a constant refractive index polymer interfacial layer squeezed in between the Novec 7500 subphase (*n* = 1.290) and water (*n* = 1.333). The interfacial refractive index and the layer thickness were varied to achieve the best possible fit of the model to the experimental data.

### X‐Ray Photoelectron Spectroscopy

Emulsions generated between Novec 7500/PFBCl (10 µg mL^−1^) and PLL aqueous solutions (in PBS, pH 10.5, at a concentration of 100 µg mL^−1^; 1/2 fluorinated oil to aqueous solution ratio) were washed nine times with deionized water and allowed to dry on silicon substrates. Note that characterization of nanosheets generated with 3 kDa PLL was not possible as emulsions formed with such nanosheets were insufficiently stable to allow this protocol to be applied. Dried protein nanosheets were washed with hexafluoroisopropanol (Sigma) and ethanol to remove any soluble residues, and finally dried again. XPS was carried out using a Nexsa X‐ray photoelectron spectrometer (XPS) system on samples prepared as for SEM characterization. A pass energy of 200 eV and a step size of 1 eV were used for survey spectra. For high energy resolution spectra, a pass energy of 50 eV and a step size of 0.1 eV were used. The spectrometer charge neutralizing system was used to compensate sample charging and the binding scale was referenced to the aliphatic component of C 1s spectra at 285.0 eV. The concentrations obtained were reported as the average percentage of that particular atom species (atomic%) at the surface of four samples (<10 nm analysis depth) without any correction. The analysis area (0.3 × 0.7 mm^2^), the angle of incidence, and the beam intensity were kept constant for all measurements.

### Mesenchymal Stem Cells (MSCs) Culture and Seeding

Bone marrow derived human mesenchymal stem cells (PromoCell) were cultured in T75 flasks in MSC growth medium (PromoCell). MSCs were harvested with 4 mL accutase‐solution (PromoCell), resuspended, then centrifuged. 5000 cells per well (resuspended in medium) were seeded on flat interfaces (per well in 24‐well plates) and cultured in an incubator (37 °C and 5% CO_2_). Half of the medium was replaced with fresh medium every two days. For passaging, 300 000 cells were seeded in a T75 flask.

### Generation of Flat Fluorinated Oil‐Culture Medium Interfaces for Monitoring of Cell Expansion

24‐well plates were plasma treated for 10 min. 500 µL of ethanol containing 10 µL trimethylamine and 10 µL trichloro (1H, 1H, 2H, 2H‐perfluorooctyl) silane were added into each well. After 24 h incubation, the solution was removed, and wells were washed with ethanol. After washing with PBS twice, 500 µL of Novec 7500 containing the desired prosurfactant at a desired concentrations (see detail of each figure) were transferred into each well. 1 mL of PBS (pH adjusted to 10.5) was carefully pipetted at the surface of the oil, followed by 1 mL of 200 µg mL^−1^ PLL solution (in pH 10.5 PBS; final concentration of 100 µg mL^−1^) and incubation for 1 h. Interfaces were washed by dilution/aspiration with PBS (pH 7.4) six times. Fibronectin adsorption was carried out by adding 20 µL of a fibronectin solution (1 mg mL^−1^) into each well, to make a final concentration of 10 µg mL^−1^, and incubated for 1 h. Functionalized interfaces were washed by dilution/aspiration with PBS (pH 7.4) four times, and then with growth medium twice.

### Preparation of Glass‐Mounted Wells for Higher Resolution Imaging

Fluorinated thin glass slide (25 × 60 mm) was attached to Sticky‐Slide 8 Well (an 8‐well bottomless µ‐slide with a self‐adhesive underside to which substrates can be mounted, Ibidi). After sterilization with 70% ethanol, wells were washed with PBS and filled with 600 µL PBS (with pH adjusted as indicated). Then 10 µL pinned droplets of fluorinated oil (Novec 7500) with the fluorinated co‐surfactant (PFBCl) at desired concentrations were deposited on top of the submerged coated glass substrate. 300 µL PBS was removed by micropipette aspiration. 300 µL of 200 µg mL^−1^ PLL solution was pipetted into each well (final concentration of 100 µg mL^−1^) and left to incubate for 1 h. Each well was washed by successive dilution/aspiration with PBS (pH 7.4, 6 times). Fibronectin adsorption was carried out by adding 6 µL of a fibronectin solution (1 mg mL^−1^) into each well (final concentration: 10 µg mL^−1^), followed by incubation at room temperature for 1 h. Finally, each well was washed by dilution/aspiration with PBS (pH 7.4) four times and then with growth medium twice.

### Viability Assay and Hoechst Staining for Cell Counting

Cell viability and proliferation on flat interfaces were assessed using a Live/Dead viability/cytotoxicity Kit (Invitrogen) and Hoechst staining. Half of the medium in each well was replaced with pre‐warmed PBS containing 2 µL Hoechst (1 mg mL^−1^ Thermofisher Scientific) and the Live/Dead staining solutions. After 30 min incubation, cells were imaged using a Leica DMI4000 fluorescence or a Leica DMi8 epifluorescence microscope. Cell counting was carried out by thresholding and watershedding nuclear images in ImageJ.

### Immunostaining

For immunostaining and imaging at higher resolution, cells were cultured on pinned droplet generated in a sticky‐slide 8 well plates (Ibidi), prepared as described above. After 24 or 48 h incubation, each well was diluted with PBS six times before samples were fixed with 8% paraformaldehyde for 10 min and diluted with PBS six times before permeabilization with 0.4% Triton X‐100 for 5 min at room temperature. Samples were blocked for 1 h (blocking buffer: PBS containing 10 vol% fetal bovine serum and 0.5 vol% gelatine), combining with tetramethyl rhodamine isothiocyanate phalloidin (1:500, Sigma‐Aldrich). Samples were subsequently incubated with primary antibodies (anti‐vinculin mouse monoclonal, Sigma‐Aldrich, 1:200 and anti‐Ki67 rabbit monoclonal, Epredia, 1:500, in blocking buffer) for 1 h at room temperature, diluted with PBS six times, then incubated with Alexa Fluor 488‐conjugated secondary antibodies (goat anti‐mouse, 1:500 in blocking buffer) and DAPI (1:500) for 1 h at room temperature. The samples were washed six times by dilution with deionized water and imaged shortly after. Immunostaining for the quantification of fibronectin adsorption was carried out directly after adsorption, without cell culture. Samples were blocked and prepared as described above prior to staining, first using a fibronectin antibody raised in rabbit (Sigma, F3648, 1:500), followed by secondary staining with an Alexa Fluor 488‐conjugated secondary antibody (donkey anti‐rabbit, 1:500 in blocking buffer). Both incubations were carried out for 1 h. As control, incubation with a mismatched Alexa Fluor 488‐conjugated secondary antibody was used (goat anti‐mouse, 1:500 in blocking buffer).

### Imaging of PLL Nanosheets Assembled at Oil Interfaces

Fluorinated glass slides (1 × 1 cm) were placed in a 24‐well plate. After sterilization with 70% ethanol, the wells were washed (twice) and then filled with 2 mL PBS (pH 10.5). 100 µL droplets of fluorinated oil (Novec 7500) with the fluorinated co‐surfactant PFBCl at a concentration of 10 µg mL^−1^ were deposited on the glass samples and formed a fluorinated oil droplet spreading over the entire substrate. Subsequently, a labelled PLL solution (2 µL, PLL‐Alexa Fluor 594 at 10 mg mL^−1^, mixed with 18 µL of PLL solution at 10 mg mL^−1^) was added to PBS to make a final PLL concentration of 100 µg mL^−1^, and the resulting interfaces were incubated for 30 min. PLL adsorption was interrupted by reducing the pH below 5, by adding a drop of 1.0 m HCl. The staining solution was then diluted with PBS (pH 7.4) eight times, prior to fluorescence imaging. Blebbistatin treatment: Myosin II was inhibited by treating cells seeded on PLL‐Alexa Fluor 596‐stabilised pinned droplets with 20 µM blebbistatin (Sigma‐Aldrich) or DMSO. Blebbistatin treatment was initiated 3 h after cell seeding to allow initial cell adhesion. After 24 h treatment, cells were fixed and imaged to qualitatively assess interface rupture due to cell contractility.

### Immuno‐Fluorescence Microscopy and Data Analysis

Fluorescence microscopy images were acquired with a Leica DMI4000B fluorescence microscopy (CTR4000 lamp; 63× 1.25 NA, oil lens; 10× 0.3 NA lens; 2.5× 0.07 NA lens; DFC300FX camera) and a Leica DMi8 epifluorescence microscope (HC PL FLUOTAR 10×/0.32 PH1; HC PL FLUOTAR 63×/1.30 Oil PH3; LEICA DFC9000 GT sCMOS camera). Confocal microscopy images were acquired with a Leica TCS SP2 confocal microscope (X‐Cite 120 LED lamp; 63× 1.40‐0.60 NA, oil lens; 10× 0.3 NA lens; DFC420C CCD camera) and a Zeiss Super resolution LSM710 ELYRA PS.1 (EC Plan‐Neofluar10×/0.3 M27; EC Plan‐Neofluar20×/0.5 M27; sCMOS camera). Cell densities were determined after thresholding and watershedding nuclei images in ImageJ. In the case of cell aggregates, cells were counted manually. To determine adhesion cell areas, images were analyzed by outlining the contour of the cell cytoskeleton (phalloidin stained) and areas were measured in ImageJ. To determine cell spreading areas, images were analyzed by thresholding and watershedding cytoskeleton images (phalloidin staining). For confocal imaging, stacks of 16 sections were scanned, with an image averaging of 2 and a line averaging of 4. 3D reconstruction and volume rendering of the stacks were performed via Imaris x64.

### Statistical Analysis

All experiments were carried out in separate experimental triplicate. Quantitative results were presented as mean values and standard errors. Statistical analysis was carried out using Origin 2019 through one‐way ANOVA with Tukey test for posthoc analysis. Significance was determined by *p < 0.05, **p < 0.01, ***p < 0.001, and n.s., non‐significant.

## Conflict of Interest

The authors declare no conflict of interest.

## Supporting information

Supporting information

## Data Availability

The data that support the findings of this study are available from the corresponding author upon reasonable request.
