# Supplementary material for: Mesenchymal Stem Cells Sense the Toughness of Nanomaterials and Interfaces
Source: Adv Healthc Mater. 2023 Feb 21;12(13):2203297. doi: 10.1002/adhm.202203297 (PMC11468436; doi:10.1002/adhm.202203297)
Supplement: Supplementary file 1 — Supporting information [file ADHM-12-2203297-s001.pdf]

ADVANCED HEALTHCARE MATERIALS

Supporting Information

for *Adv. Healthcare Mater.*, DOI 10.1002/adhm.202203297

Mesenchymal Stem Cells Sense the Toughness of Nanomaterials and Interfaces

*Lihui Peng, Carlos Matellan, Minerva Bosch-Fortea, Jordi Gonzalez-Molina, Matteo Frigerio, Stefan Salentinig, Armando del Rio Hernandez and Julien E. Gautrot**

Supplementary Information

Mesenchymal Stem Cells Sense the Toughness of Nanomaterials and Interfaces

Lihui Peng[1,2], Carlos Matellan[3], Minerva Bosch-Fortea[1,2], Jordi Gonzalez-Molina[1,2], Matteo Frigerio[4], Stefan Salentinig[4], Armando del Rio Hernandez[3] and Julien E. Gautrot[1,2*]

[1] Institute of Bioengineering and [2]School of Engineering and Materials Science, Queen Mary, University of London, Mile End Road, London, E1 4NS, UK.

[3] Cellular and Molecular Biomechanical laboratory, Department of Bioengineering, Imperial College London, SW7 2AZ, UK.

[4] Department of Chemistry, University of Fribourg, Chemin du Musée 9, Fribourg 1700, Switzerland.

[*]Corresponding author. j.gautrot@qmul.ac.uk

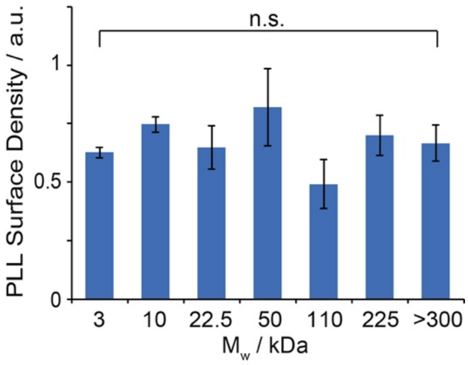

Supplementary Figure S1. Quantification of PLL density within nanosheets assembled at the Novec 7500-water interfaces (Novec 7500 containing 10 µg/mL PFBC; aqueous solution is PBS with pH adjusted to 10.5). PLL (tagged with fluorescent dye) with different M_w (3, 10, 22.5, 50, 110, 225 and >300 kDa) was introduced to make a final solution with a concentration of 100 µg/mL. One-way ANOVA; n.s., non significant.

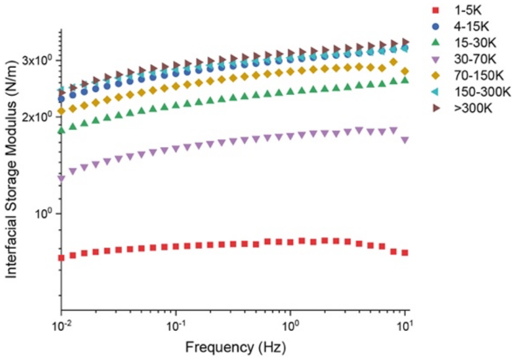

Supplementary Figure S2. Frequency sweep profiles of interfaces consisting of PLL nanosheets assembled at the Novec 7500-water interfaces (Novec 7500 containing 10 µg/mL PFBC; aqueous solution is PBS with pH adjusted to 10.5; strain of 10^{-3} rad). PLL with different M_w (3, 10, 22.5, 50, 110, 225 and >300 kDa) was introduced to make a final solution with a concentration of 100 µg/mL.

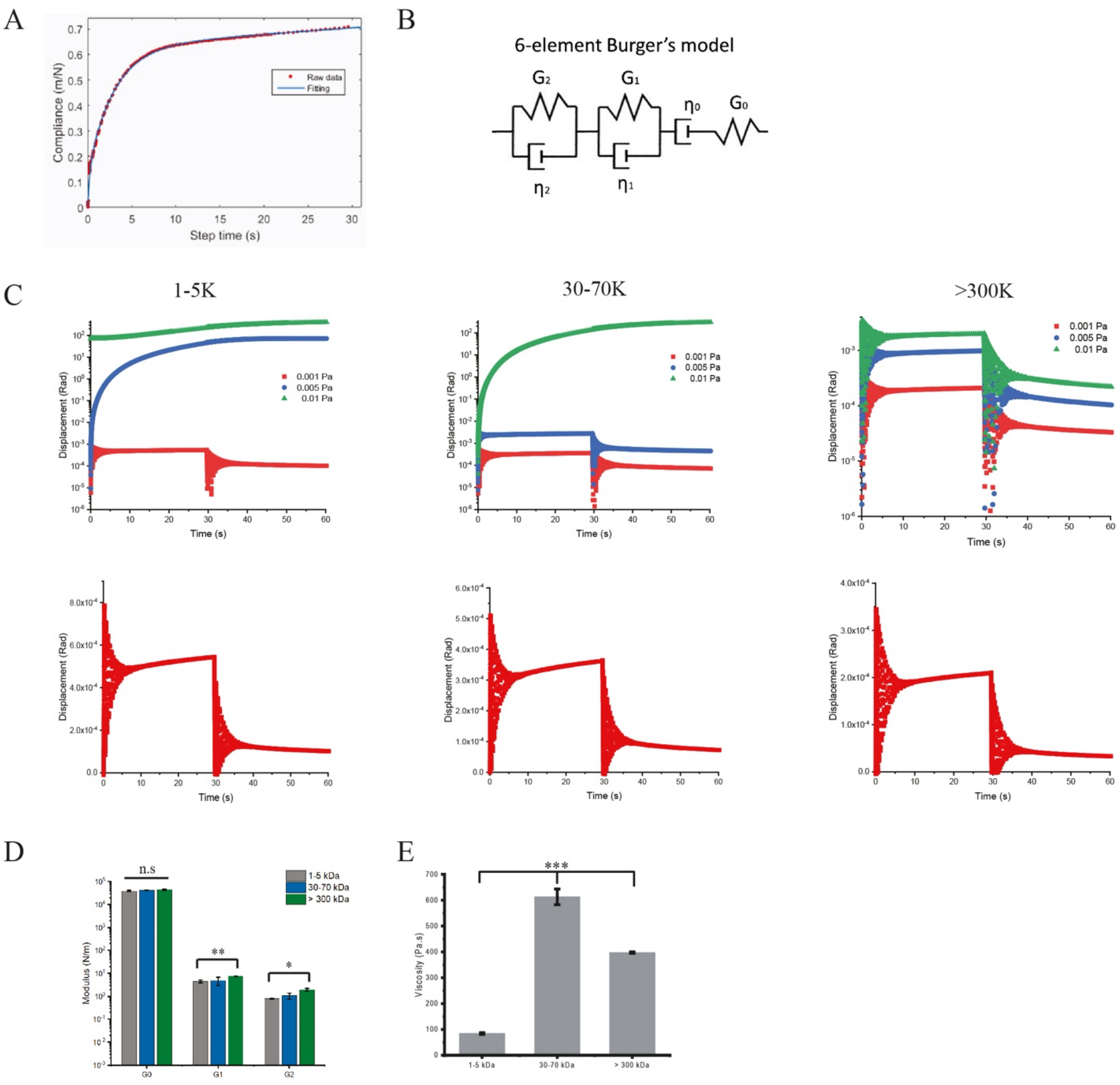

Supplementary Figure S3. Interfacial creep experiments carried out at the Novec 7500/PBS interfaces (Novec supplemented with 10 μg/mL PFBC; PBS with pH adjusted to 10.5, containing 100 μg/mL PLL at different M_w (3, 50 and >300 kDa). A-B) Representative creep trace fitted with a 6-element Burger's model and schematic illustration of a 6-element Burger's model. It consists of two Kelvin–Voigt elements (a spring and a dashpot in parallel) in series with a Maxwell element (a dashpot and a spring in series). C) Representative creep and recovery curves at different oscillation stresses (top) and at stress of 0.001 Pa alone (bottom). D-E) The viscoelasticity parameters (D, elastic modulus, G0, G1 and G2, top; E, and viscosity η) extracted from interfacial creep experiments, fitted with 6-element Burger's model. Error bars are s.e.m.; n=3. One-way ANOVA; n.s., non significant; *, $p<0.05$; **, $p<0.01$; ***, $p<0.001$.

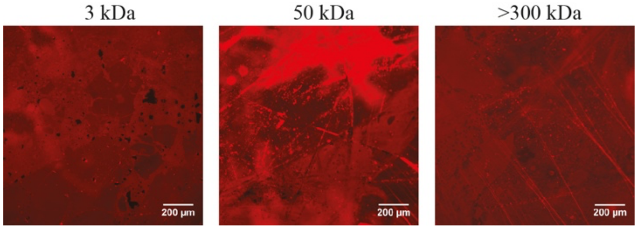

Supplementary Figure S4. Epifluorescence images of PLL nanosheets harvested from liquid-liquid interfaces on mica substrates, via Langmuir Blodgett deposition and confirming that large areas are fully covered by nanosheets.

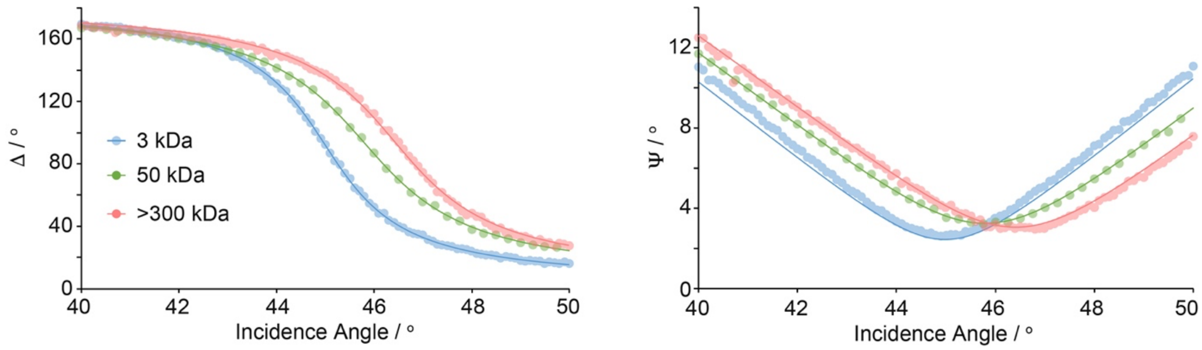

Supplementary Figure S5. In situ ellipsometry data with their corresponding fits obtained for PLL nanosheets (3, 50 and >300 kDa) assembled at Novec 7500/PBS interfaces, in the presence of PFBC (10 µg/mL). The concentration of PLL was 100 µg/mL, and the pH was 10.5.

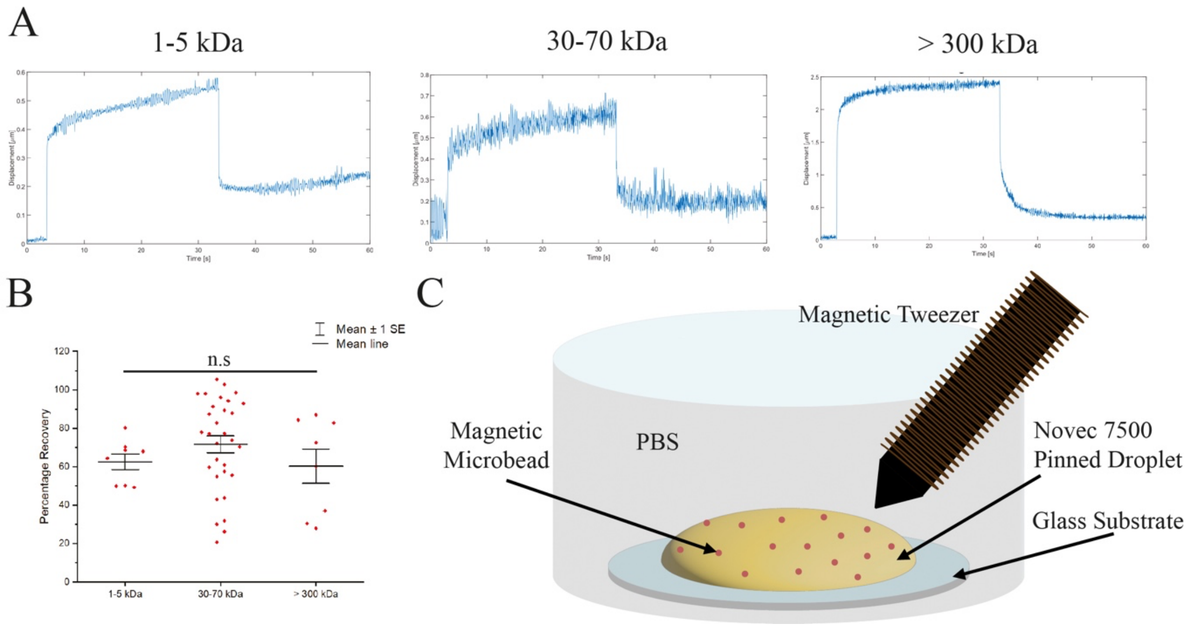

Supplementary Figure S6. Interfacial microrheology creep experiments using magnetic tweezers. Magnetic beads were allowed to adhere to nanosheets formed at the interface between Novec 7500 containing 10 µg/mL PFBC and PBS (pH 10.5) containing 100 µg/mL PLL at different M_w (3, 50 and >300 kDa). (A) Representative creep and recovery curves and (B) corresponding percentage recovery extracted from fitting the data to a 6-element Burger's model. Error bars are s.e.m.; n⩾7. (C) Schematic representation of the magnetic tweezer experimental set up for magnetic tweezer in this study. One-way ANOVA; n.s., non significant.

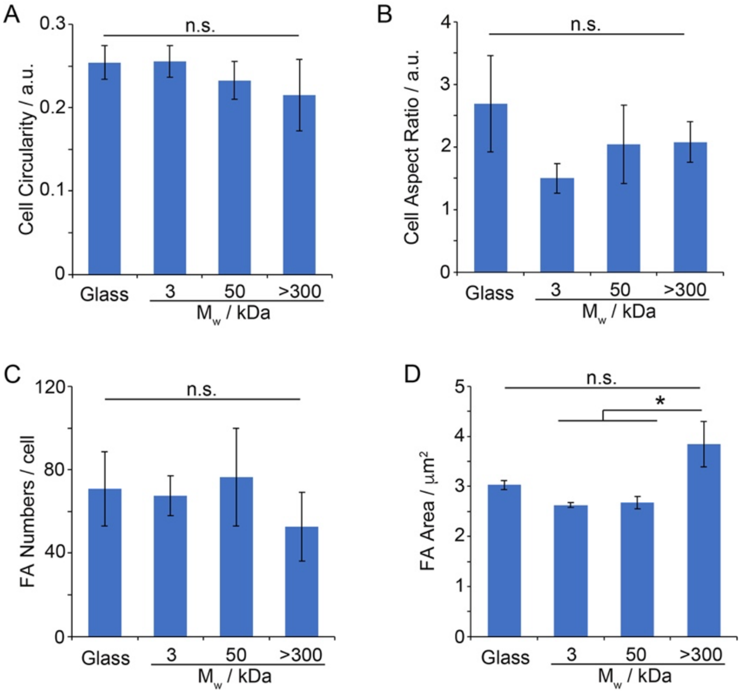

Supplementary Figure S7. Quantification of cell circularity (A) and aspect ratio (B), as well as number of focal adhesions per cell (C) and focal adhesion areas (D) measured for MSCs spreading on TPS and Novec 7500 oil interfaces, stabilised by PLL nanosheets (24 h). Error bars are s.e.m,; n = 3. Detail of interfaces: Novec 7500 containing 10 µg/mL PFBC; aqueous solution is PBS with pH adjusted to 10.5; PLL with different M_w (3, 50 and >300 kDa) at a final concentration of 100 µg/mL. One-way ANOVA; n.s., non significant; *, $p<0.05$.

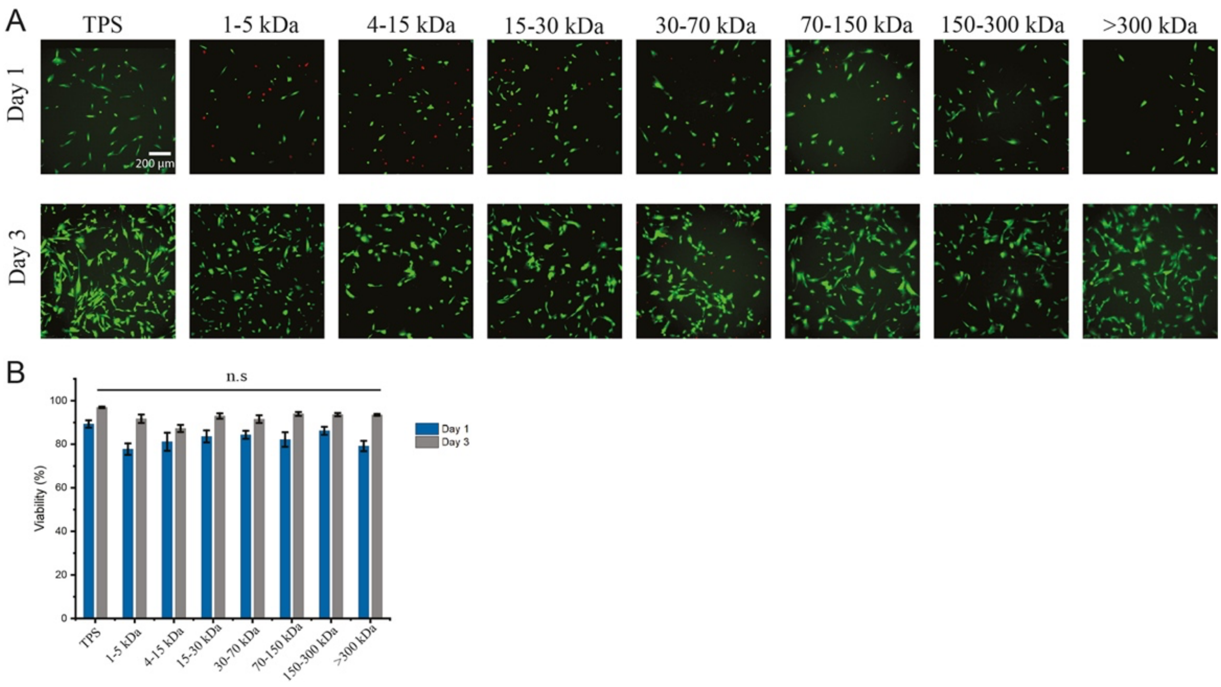

Supplementary Figure S8. A) Epifluorescence microscopy images of MSCs cultured on TPS and Novec 7500 oil interfaces, stabilised by PLL nanosheets (days 1 and 3). Live/Dead staining: green, live cells; red, dead cells. B) Quantification of corresponding cell viabilities. Error bars are s.e.m,; n = 3. Detail of interfaces: Novec 7500 containing 10 µg/mL PFBC; aqueous solution is PBS with pH adjusted to 10.5; PLL with different M_w (3, 10, 22.5, 50, 110, 225 and >300 kDa) at a final concentration of 100 µg/mL. One-way ANOVA; n.s., non significant.

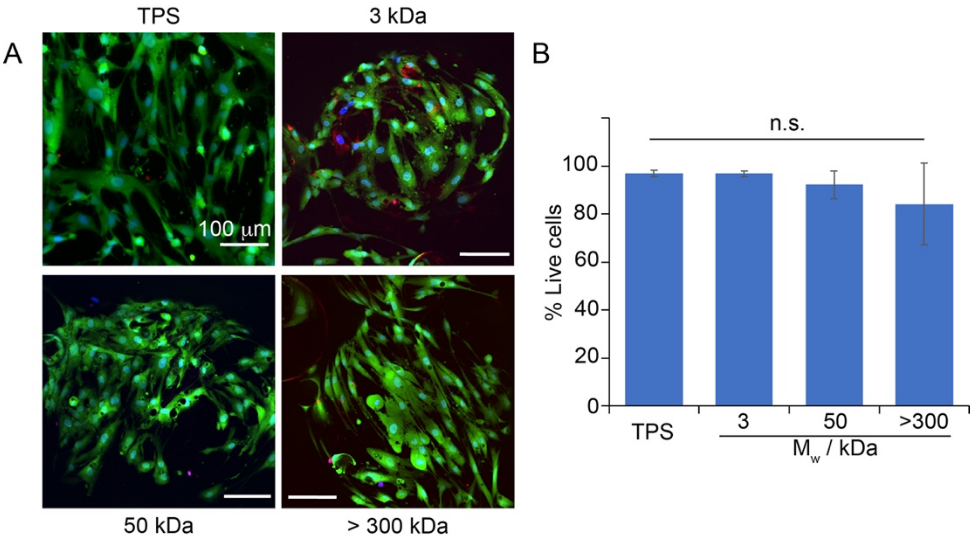

Supplementary Figure S9. A) Epifluorescence microscopy images of MSCs cultured on TPS and Novec 7500 oil interfaces, stabilised by PLL nanosheets (cultured for 6 days). Live/Dead staining: green, live cells; red, dead cells. B) Quantification of corresponding cell viabilities. Error bars are s.e.m,; n = 3. Detail of interfaces: Novec 7500 containing 10 µg/mL PFBC; aqueous solution is PBS with pH adjusted to 10.5; PLL with different M_w (3, 50 and >300 kDa) at a final concentration of 100 µg/mL. One-way ANOVA; n.s., non significant.

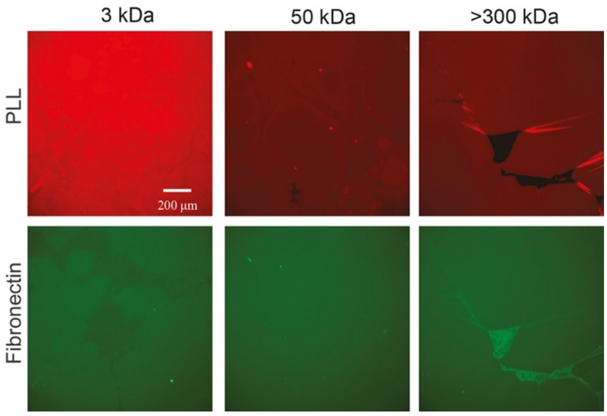

Supplementary Figure S10. Epifluorescence images of PLL and fibronectin forming PLL/fibronectin nanosheets assembled at the interfaces between Novec 7500 containing 10 µg/mL PFBC and PBS (pH adjusted to 10.5; PLL with different M_w (3, 10, 22.5, 50, 110, 225 and >300 kDa) at a final concentration of 100 µg/mL.

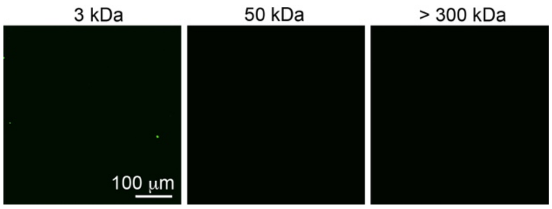

Supplementary Figure S11. Control of epifluorescence images of PLL/fibronectin nanosheets assembled at the interfaces between Novec 7500 containing 10 µg/mL PFBC and PBS, stained with mismatched secondary antibody (pH adjusted to 10.5; PLL with different M_w (3, 50 and >300 kDa) at a final concentration of 100 µg/mL.

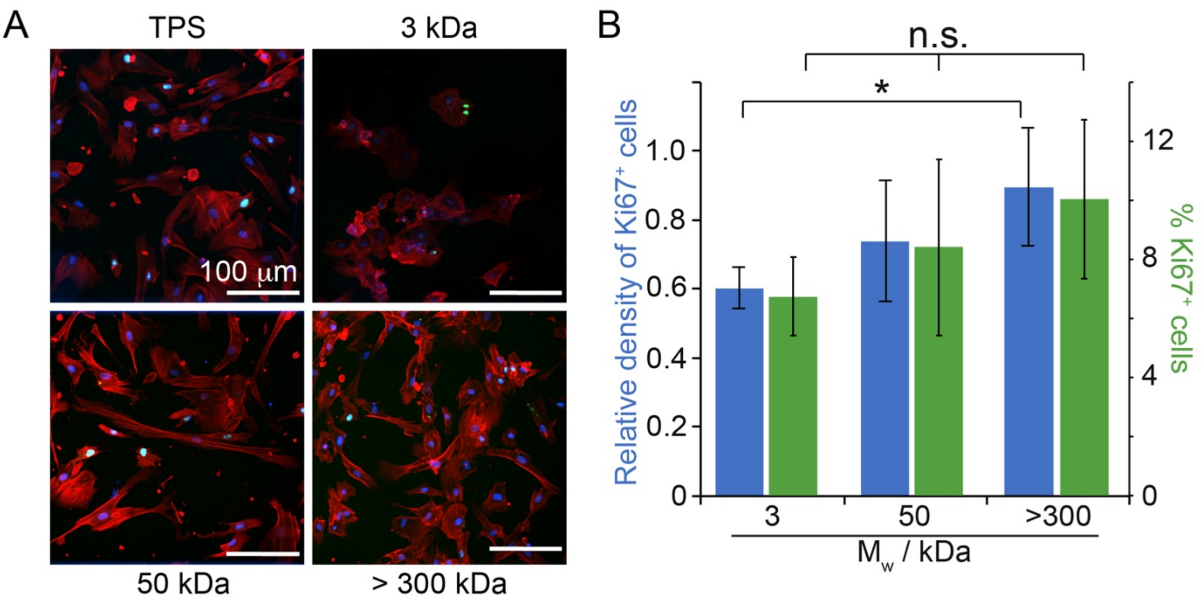

Supplementary Figure S12. A) Ki67 expression by MSCs cultured on PLL/FN nanosheets assembled at the surface of Novec 7500. Epifluorescence microscopy images of MSCs cultured at the interfaces for 6 days. Detail of interfaces: Novec 7500 containing 10 µg/mL PFBC; aqueous solution is PBS with pH adjusted to 10.5; PLL with different M_w (3, 50 and 225 kDa) at a final concentration of 100 µg/mL. Red, F-actin; Green, Ki67; Blue, DAPI. B) Corresponding quantification of $Ki67^+$ cells, relative to the levels of expression observed on TPS. Error bars are s.e.m,; n = 3. One-way ANOVA; n.s., not significant; *, p<0.05.

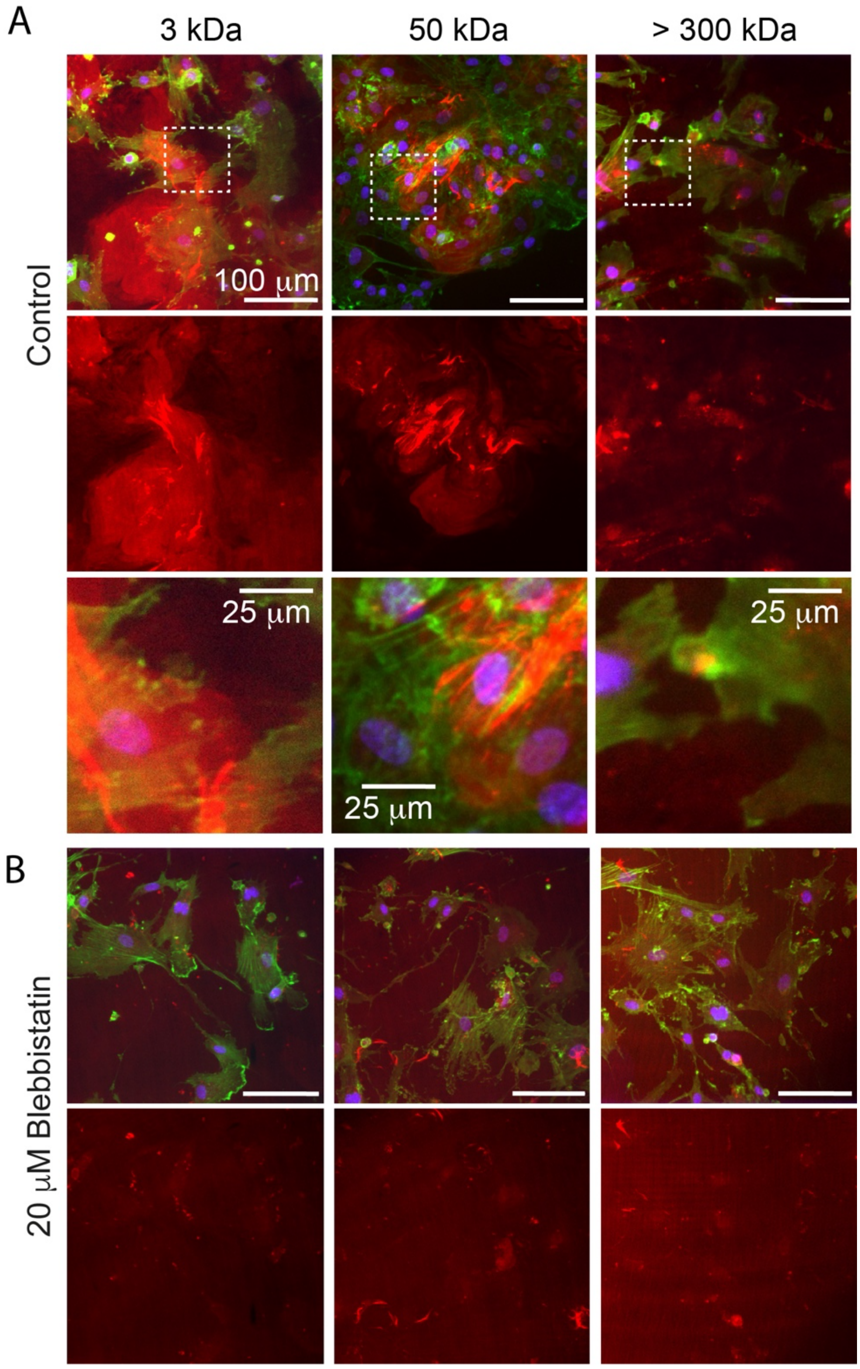

Supplementary Figure S13. MSCs induce the wrinkling and aggregation of PLL/FN nanosheets assembled at the surface of Novec 7500, without (A) and with (B) blebbistatin (supplemented at 20 μM). Epifluorescence microscopy images of PLL nanosheets after culture of MSCs at their surface for 48 h. Detail of interfaces: Novec 7500 containing 10 μg/mL PFBC; aqueous solution is PBS with pH adjusted to 10.5; PLL with different M_w (3, 50 and 225 kDa) at a final concentration of 100 μg/mL. Red, PLL; Green, F-actin; Blue, DAPI.
